# Perspectives on Inclusive Education of Preschool Children with Autism Spectrum Disorders and Other Developmental Disabilities in Iran

**DOI:** 10.3390/ijerph15102307

**Published:** 2018-10-20

**Authors:** Sayyed Ali Samadi, Roy McConkey

**Affiliations:** 1 Institute of Nursing and Health Research, Ulster University, Co. Antrim BT37 0QB, UK; r.mcconkey@ulster.ac.uk; 2 Northern Ireland and the Medical Sciences Division, Academic Center for Educational, Cultural and Research (ACECR), Shahid Beheshti University, Tehran 1985717443, Iran

**Keywords:** inclusive education, developmental disabilities, autism, parents’ attitudes, teachers’ attitudes

## Abstract

*Background*: Iranian children with disabilities invariably attend special schools and many may be excluded from education entirely. Information on preschool education is limited but probably mirrors the situation in schools. There is a lack of information in terms of parental preferences for schooling and teachers’ experiences of inclusion in Iran. *Method*: Two feasibility studies were undertaken; one with 89 parents of children with autism or intellectual disabilities, and another with the head teachers of two private kindergartens. *Results*: Two-thirds of parents favored inclusive schools; most parents whose children had autism or were verbally proficient were in favor of their child attending ordinary schools, even if their child had been placed in a specialist preschool facility. The head teachers justified inclusion in terms of children’s rights but identified three main challenges: coping with the diverse level of functioning, the need for special devices and training of teachers, and challenging the negative reactions of parents of non-disabled children. *Conclusions*: Further exploration of the views of those who have experienced inclusion would further challenge existing practices. Moreover, the training and preparation of teachers is key to reforming schools. However, wider social values and beliefs towards disabilities also need to change.

## 1. Introduction

In Iran, the predominant attitude is that children with special needs cannot attend ordinary schools and are unable to sit next to their typically developing peers. However, education is a pathway to citizenship and a basic right of children [1] regardless of their developmental course. Instead, students with special needs attend mostly special schools under the aegis of the Iranian Special Education Organization (ISEO). In 1997, proposals for inclusive education were prepared; nevertheless, these recommendations are yet to be implemented [2].

All Iranian children are required to go through a national screening system at 6 years of age to assess their suitability for registration in the first grade of primary school. The program consists of a dental checkup and assessments of the child’s vision, hearing, height, and weight undertaken by a general physician and another health professional. The child’s communication level and readiness for education are assessed by an educational counsellor using a specially devised scale called “The first step.” Each assessor sees every child individually. Those children who do not pass the screening are referred for a professional evaluation. The outcomes are documented in the children’s Health Identification Booklet, which is given to the parents and is necessary for the child to enter the first grade of elementary education.

In 2014, 1,233,671 children underwent evaluation at 862 bases (plus 17 mobile bases for those nomadic tribes that move around different provinces) located in 32 national provinces [3]. A total of 1,078,421 children (87%) were deemed suitable to attend ordinary schools, as no problems were detected. The remaining 13% of children were suspected of having problems and were referred for specialist evaluation by psychologists, rehabilitation professionals, and specialist assessors employed by the ISEO. This group included children with developmental delays and intellectual disabilities as well as those who were not deemed ready for formal education. Over 90% of these children were placed in special schools. For example, in the 2014 national screening program, there were only 1380 (10%) students with motor, visual, or hearing disabilities in the inclusive education system. For some, the extra assistance provided by special schools is intended to enable them to attend ordinary schools, although the numbers transferring from special to ordinary schools are not known.

Table 1 presents the overall population of students with special needs located in ordinary schools, namely 49,553; the majority of these students have specific learning disabilities that tend to be identified once they commence schooling. However, this number is only 0.72% of the total number of students at the primary level in Iran (*N* = 6,850,000).

These figures confirm the prevailing attitude towards inclusion in Iran. For example, the Ministry of Education states that inclusion “means including children with various special educational needs in regular school settings. There are, however, limitations on such special needs, such as children with obvious mental disabilities. Down syndrome children are natural candidates for this category” [4].

For preschoolers with special needs, the situation is different. Preschools and kindergartens are under the supervision of the Iranian Social Welfare Organization (ISWO), and accepting children with special needs depends on the head teachers’ decision and the dominant educational philosophy at the center. However, no figures are available for the number of children under 6 years enrolled in preschools and kindergartens, or the proportion with special needs. Similarly, children with special needs may be enrolled in private preschools but, again, the numbers are not collected centrally. The ISWO also provides pre-vocational centers for school-age children with more severe disabilities who are deemed not to benefit from education; however, adult persons are also enrolled in these centers, and information is only available on the total numbers of people attending these centers: some 500,000 in 2015, of whom the largest proportion (92%) have physical disabilities. Hence, it is likely that many children with more severe intellectual disabilities remain at home.

Thus, the international trend towards greater inclusion of children in ordinary educational settings is yet to impact on Iran, where most children attend special schools, and those with more severe disabilities are excluded from the education system, with some attending centers managed by the Iranian Social Welfare Organization. Nevertheless, a study in one Iranian province suggested that managers of inclusive education projects were more hopeful of success than were teachers and clinical “experts” such as psychologists and rehabilitation specialists. They perceived that students with special needs would benefit from increased social opportunities and that all students would receive more individualized teaching [5].

Many preschool centers are not willing to take young children with disabilities into their centers. Some of the reasons for this include lack of preparation of guardians to meet children’s basic needs, inadequate buildings, shortage of materials and equipment, and the belief that these children need “professional assistance.” Equally, parents may be reluctant to send their children with special needs to preschool centers perhaps because of feelings of shame or fear that their needs would not be met, they would be teased or bullied by other children, or would even negatively impact other non-disabled children [6]. These are common feelings reported in other societies [7].

The impact of child developmental disabilities diagnosis on parents and its associated strains have been documented in Iran [8,9], and the reported findings are broadly similar to those in other cultures; there are indications that those children with Developmental Disabilities (DD), such as children with autism spectrum disorders (ASD), present distinct challenges to their parents.

In many countries, associations of parents of children with disabilities and disabled persons organizations have been in the forefront advocating for greater inclusion within society and working to change the attitudes of families and the general public [10]. However, specific efforts are needed to promote such organizations in Iran [11].

## 2. Feasibility Study

One of the main lobby groups internationally campaigning for greater inclusion has been the parents of children with intellectual disabilities. This builds on the increasing emphasis placed by education systems on the value of parental involvement in the education of children [12]. However, in Iran, few studies have investigated parents’ views on their child’s school placement, which reflects the overall lack of parent advocacy within the education system. Hence, a feasibility study was undertaken to ascertain how parental views on educational provision for children with intellectual disabilities and autism spectrum disorders (ASD) could be sought. This information was complemented by interviews with two head teachers of kindergartens with experience in enrolling children with these disabilities in their centers. Again, this aimed to determine the feasibility of this approach in gaining insight into how opportunities for inclusive schooling might be advanced in Iran.

## 3. Methods

### 3.1. Participants

A self-administered questionnaire was sent to 112 parents of preschool children with developmental disabilities (aged 2 to 5 years) who were already registered at school by the ISEO and the ISWO central bureau in Tehran. Families came from different districts of the city and had different social backgrounds. Information was collected on the child and family characteristics as well as their preferred future educational placement for the child. Only one parent per family was asked to respond. Out of the 112 parents that received questionnaires, 89 parents replied (79% response rate); of these, 36 (40%) were parents of children with intellectual disabilities (20 mothers and 16 fathers) and 53 (60%) were parents of children with autism spectrum disorders (25 mothers and 28 fathers). Further details of the participants are presented in Table 2.

### 3.2. Parental Questionnaire

The self-administered questionnaire consisted of three main sections. The first section requested demographic information such as parental age, education, and main wage earner (three questions). The second section requested general information about the child, such as age, gender, order of birth, diagnosis, verbal ability and behavioral problems, homeschooling, and out-of-home services (such as clinical services or daycare centers) (eight questions). In the third section, the parents selected their desired educational settings from three options (special school, inclusive schools. and undecided).

### 3.3. Head Teachers Interview

The head teachers (HT) of two private kindergartens (one in Mahabad (HT1) and another in Tehran (HT2)) known for accepting children with different developmental disabilities were interviewed about their experiences regarding accepting children with special needs at their centers. Overall, 12 children with special needs were registered in the two kindergartens. Both interviews were performed at the head teachers’ office in the kindergarten. A semi-structured interview method was used to explore the teachers’ attitudes toward inclusive education, its challenges, and how they might be addressed. Open-ended questions were used to encourage the interviewee to elaborate freely and reflectively on the topics posed. At the end of each interview, the participants were asked if they wished to say anything else about the topic [13]. Two raters independently examined the interview transcripts and used thematic content analysis to identify the main themes common to both interviews. A 95%-consensus between the two independent raters was found on the themes on four pages of the summarized interviews.

## 4. Findings

### 4.1. Parents’ Views

Of the 89 parents, 58 (65%) favored inclusive schools, 30 (34%) special schools, and 1 was undecided. Parents of children with autism were more likely to favor inclusive schools than were parents of children with intellectual disabilities (82% vs. 41%, Chi Sq 15.4, *p* < 0.001). Moreover, parents whose children were verbally fluent opted for inclusion (88% vs. 37% of those not verbally fluent, Chi Sq 25.1, *p* < 0.001), as did those whose children had behavioral challenges (77% vs. 51% of those with no behavioral challenges, Chi Sq 6.02, *p* < 0.05).

Additionally, parents whose children already attended a specialist education center were more in favor of inclusion than those parents whose children were not in an educational setting (88% vs. 56%, Chi Sq 8.35, *p* < 0.005), although this is confounded with the child’s disability, as significantly more children with ASD attended a special center than did those with intellectual disability (89% vs. 47%, Chi Sq 13.9, *p* < 0.001) (Table 3).

Other variables such a parents’ gender (*p* = 0.910), age (*p* = 0.715), and educational level (*p* = 0.883) did not affect parents’ preferences for future schooling.

### 4.2. Head Teachers’ Views

Overall, the head teachers spoke positively about having children with special needs in their kindergartens and gave reasons for why they had enrolled these children.

“Some parents of typically developing children raised objections when they heard that we have special children. Some even threaten to withdraw their child from here. They are afraid of the negative impact of these children on their own children. But I think that we should prepare children for real life. I have no authority to choose my neighbors, so I may have neighbors with different abilities and I have to learn how to get along with them. We provide a real-world situation here, although it is difficult. Some think (that we do) it just for money!” (HT1).

“We should take care of all the children regardless of their development style. Typically developing children have their own problems. This is similar to children we call special but there was new staff here in our kindergarten who were not so comfortable with having a student with special needs in the class. This was because they have never had this experience. But as soon as they accept the child with special needs, they will find the way to handle them during their time with us here.” (HT2).

When the challenges of having children with developmental disabilities in the kindergartens were probed, three main themes emerged.

#### 4.2.1. Concerns about Level of Functioning of Children with Special Needs

The head teachers felt that there were pre-requisite skills that the children needed to have for their successful placement in ordinary educational settings. However, these mostly referred to the child’s personal care needs rather than verbal abilities, which the parents’ survey had identified. However, behavioral difficulties were a further reason for excluding a child, even though the parents of these children felt such settings would be preferable.

“There are some skills that the child should have. I mean things like the ability to control bowel and bladder. Some other things are also essential, such as the ability to eat and some degree of walking ability…I am not sure but sometimes these children are afraid of being among other children and therefore they are not good candidates for being registered.” (HT1).

“At the beginning, we claimed that we accept all the children regardless of their level of abilities. You know that we are officially free to accept and reject children and it is up to us to decide. No rules and regulation for us, but we knew that there should be some skills, and it depends on the different cases, sometimes everything seems OK with them but behavioral problems do not let us to have them. So we keep them temporary, for 1 week, and then we decide about accepting or rejecting them.” (HT2).

#### 4.2.2. Concerns about Additional Services such as Special Devices and Staff Training

The head teachers noted the need for the kindergartens to have access to special equipment in order to assist the children, and for the staff to have access to training to help them adapt their teaching to the child’s needs.

“We need special equipment, sometimes parents will help with this and bring what we need but sometimes, because we are not ready for every unexpected situation, we feel that we desperately need something to assist us to be more useful. These children need more supervision. Sometimes our staff face situations they are not ready for. If I am asked to mention our needs in this regard, I will mention training courses for my staff. Training on children with special needs.” (HT1).

“There are some regulations to make our environment safe and we have to consider all those, so we are not totally ready for accepting them [children with special needs]. I mean steps and playground. Some children require special chairs and tables in the class. But we do our best because we want to accept them. Their parents have no other choice and somebody should care about this.” (HT2).

#### 4.2.3. Reactions of Other Parents

The head teachers were conscious that other parents might react negatively to having a child with special needs included with their child.

“Parents of typically developing children are not happy with having children with special needs seating near their child. They say that their child might learn strange behaviors that these children show. We always consider the prejudice of these parents and try to calm them down. In reality, we did not have any conflicts between children. Of course, we are watching them all the time but I mean children have no problems with each other. We as grownups have problems with it.” (HT1).

“We have a problem with parents as well. Both groups of parents. Parents of special children because they ask for excessive assistance; they are so concerned about the children being away from them. The problem with parents of typically developing children is that they blame children with special needs for all their child’s misbehavior.” (HT2).

## 5. Discussion and Implications

The data from these feasibility studies may not be representative of the views held by the wider population of Iranian parents whose children have developmental disabilities or head teachers in preschool educational settings. However, both studies illustrate approaches that could be used to elicit the opinions of these key stakeholders, thereby providing support for widening opportunities for inclusive education in Iran.

According to the Iranian Education Policy Act (1980) [3], after the Islamic revolution, parents are the major decision-makers for their child’s education rather than the authorities. Still, parents’ preferences for children school placement are seldom considered to date and few realistic choices are offered to them. In this study, most parents of children with autism who were verbally proficient were in favor of their child attending ordinary schools even though they had been placed in specialist preschool facilities. These experiences may have given the parents greater hope that their child could cope in ordinary school settings. These placements would also moderate the stigma they may feel regarding their child’s disability [14]. Likewise, some parents of children with intellectual disability aspired for their child to attend ordinary schools, although these parents were more disadvantaged, as their children were less likely to have received any preschool education.

The head teachers were realistic with respect to the challenges that kindergartens and schools will face in including children with developmental disabilities. In particular, they noted the need for adapting buildings, the provision of specialist aids and, crucially, the training and support of teachers. Similar conclusions have been reported internationally [15,16]. The consequences of these pessimistic beliefs are that most preschool children with disabilities spend their days at home often in un-stimulating environments.

However, these preschools had found ways of successfully including children with developmental disabilities, and their experiences could be incorporated into the wider training of teachers working in preschool settings in particular. Their insights would also be of benefit to teachers in primary schools. In addition, there is cumulative international experience regarding training teachers for inclusion [17].

A more daunting challenge is to change the wider societal attitudes toward children with disabilities, expressed here in the reactions of parents of non-disabled children toward including children with disabilities in the two kindergartens. In part, these attitudes need to be tackled by kindergartens as the head teachers reported; however, their efforts need to be matched by efforts to educate the wider Iranian public about disability and the rights of children to equal opportunities. Internationally, disabled activists and parent associations have taken a leading position doing this [18], but these initiatives are still in their infancy in Iran. However, the Iranian government has ratified the United Nations Convention on the Rights of the Child, albeit with the reservation that “If the text of the Convention is or becomes incompatible with the domestic laws and Islamic standards at any time or in any case, the Government of the Islamic Republic shall not abide by it.” Likewise, Iran ratified the UN Convention on the Rights of Persons with Disabilities in October 2009 but with a similar reservation. Both Conventions highlight the right to education of all children, which does not appear to be in conflict with Iranian domestic laws or Islamic standards. Thus, the wider political and religious contexts would appear supportive of the greater inclusion of children with disabilities in ordinary schools. A policy of inclusive education is likely to prove the most efficient and speedy means for ensuring access to education of children with disabilities nationally and in more rural provinces especially.

The remit of the Iranian Special Education Organization includes curriculum issues, the use of assistive technology and resources, and the linking of special and inclusive schools. However, the ISEO will need to make a substantial investment in teacher recruitment and training, especially with respect to children with developmental disabilities. Moreover, the dearth of trained rehabilitation and education professionals in rural areas especially needs to be addressed.

In summary, educational inclusion in Iran is presently limited to pupils in certain categories and at a certain age. The views and experiences of parents and teachers who have already started to accept inclusion and who seek to promote it need to be further explored. The training and preparation of teachers is key to reforming schools. However, wider social and cultural values and beliefs towards disabilities in general also need to change. Greater advocacy by parents and disabled activists for inclusive education is needed.

In summary, the services described in the present study have some benefits for disabled children and their families, but need to be more extensive and comprehensive, start earlier [19], and cover all special need groups in urban and rural areas. The main focus should be on the demands and desires of persons with special needs and their families [20]. Thus, services should be family-based and focus on the needs and desires of the disabled persons and their families. Service providers and planners must consider the needs of all the groups within the disabled population. Greater focus on localities rather than central planning would assist with this. The degree to which preschool services are successful in including children with special needs in their programs should be considered as an important indicator of the overall quality of early childhood education. Because the concept of inclusion is new in Iran, only time might reveal whether the legislative rhetoric of commitment to inclusion will be translated into practice. Moreover, the present focus on having specialist educators of children with intellectual, visual, and hearing impairments has also impeded the inclusion process. Unfortunately, even parents of children with different types of disabilities ask for special schools, textbooks, and other special facilities.

The goal of inclusion raises wider social themes such as greater awareness of disabilities in society, opportunities for early education interventions, involvement of families with school, and teacher education. These issues are particularly urgent in rural areas, which lack trained rehabilitation and education professionals. A policy for inclusive education is likely to prove the most efficient and speedy means of meeting this challenge.

## Figures and Tables

**Table 1:** Number and percentage of students with special needs in inclusive education in 2014–2015 academic year.

Disability Type	Number of Students and Percentage of Total
Behavioral problems (including 29 students with autism)	1536 (3%)
Physical motor disabilities	5016 (10%)
Visual impairment	2339 (5%)
Hearing impairment	5763 (12%)
Specific learning disabilities *	34,899 (70%)
Total	49,553

* The Specific learning disabilities groups of students are not screened in the national screening program but are diagnosed after registration at the schools.

**Table 2:** Demographic information of parents and children with autism spectrum disorders (ASD) and intellectual disability (ID) (*N* = 89).

**Children**
Gender: 73 boys (82%), 16 girls (18%)
Impairment: intellectual disability 36 (40%), ASD 53 (60%)
Ages: 2 years 5 (3%), 3 years 30 (34%), 4 years 27 (30%), 5 years 27 (30%)
Communication: verbal 51 (57%), non-verbal 38 (43%)
Behavioral problems: yes 52 (58%), no 32 (42%)
Attending special educational centers: yes 27 (30%), no 62 (70%)
**Parents**
Gender: mothers 45 (51%), fathers 44 (49%)
Age: under 30 years 12 (13%), 30–39 years 30 (34%), 40–49 years 40 (45%), 50+ years 7 (8%)
Education: pre-high school 21 (24%), high school 27 (30%), university 41 (46%)
Wage earner: father 72 (81%), mother 8 (9%), both 9 (10%)

**Table 3:** Parental future schooling preferences for their children with special needs.

Variable	Chi Sq	*p*	*N*
Children diagnosis	15.4	0.001	89
Children verbal ability	25.1	0.001	89
Children behavioral problems	6.2	0.05	89
Previous experience with special education	8.35	0.005	89
Children diagnosis and experience with special education	13.9	0.001	89
